# Morphology beyond the morpheme: distributed processing effects in the acoustic realization of German word-final clusters

**DOI:** 10.3389/fnhum.2026.1924343

**Published:** 2026-10-06

**Authors:** Chiara Celata, Martina Rossi

**Affiliations:** Dipartimento di Studi Umanistici (DISTUM), Università degli Studi di Urbino Carlo Bo, Urbino, Italy

**Keywords:** morpho-phonetics, acoustic reduction, consonant clusters, inflection, German

## Abstract

This study investigates how morphological structure shapes phonetic realization in German word-final /Cst/ clusters, with a focus on form-meaning mapping strategies in elicited speech. Phonologically identical clusters are compared across simplex words and inflected verb forms, where /st/ encodes a grammatical morpheme. We analyse duration, intensity, and spectral center of gravity (COG) for clusters, individual segments, and adjacent vowels, modeling their variation with linear mixed-effects models. Rather than yielding uniform phonetic shifts, morphology-related effects emerge as context-sensitive patterns modulated by lexical and phonetic factors. Temporal effects primarily take the form of durational reduction in of morphemic segments, but they extend to surrounding phonetic material, where they manifest as lengthening. In addition, increased intensity and spectral sharpening are associated with morphemic shortening, calling for a more comprehensive interpretation of phonetic reduction and its relationship with morphological function. Overall, the results are in line with approaches to speech production that move beyond a strictly modular view and posit dynamic interactions between morphological and phonetic information within the cognitive system.

## 1 Introduction

A growing body of research shows that morphology co-determines segments' acoustic properties. Studies on English, German, Dutch and a few other languages suggest that morphological information provides input to the articulatory gestures. For instance, systematic variations in the acoustic duration of word-final /s/ in English have been shown to correlate with its morphological function, whether cliticized *has* or *is*, suffixal plural (as in *cats*), or non-affixal (as in *bus*): morphemic word-final /s/ is shorter than non-morphemic /s/ (Plag et al., 2017; Schmitz et al., 2021; Plag et al., 2020). The same effect has been found for German plural /s/ compared to non-affixal word-final /s/ (Schmitz and Baer-Henney, 2024). Evidence is accumulating across an increasingly wider range of typologically diverse languages. In Bora, a predominantly agglutinative Amazonian language, /va/ affixes are shorter than homophonous non-morphemic /va/ sequences (Seifart, 2025); in Italian, a pre-dominantly fusional language, /ino/-suffixed tokens are shorter when the derivative is more transparent and segmentable (Rossi et al., 2026).

Understanding how morphological information shapes phonetic realization bears directly on broader questions concerning the cognitive architecture of language. The present study does not aim to adjudicate between competing architectural models directly. Rather, it examines whether morphological structure systematically contributes to shaping the fine-grained acoustic properties of speech, a condition that would suggest that phonetic encoding cannot be regarded as fully independent from higher-level linguistic representations.

To assess how such interactions manifest themselves in spoken language, it is necessary to consider the growing body of evidence on the phonetic effects of morphological structure. One of the central questions in this literature concerns the direction of morpho-phonetic effects, namely whether morphological structure is associated with phonetic reduction as just reported for English /s/, Bora /va/, and Italian /ino/, or rather with phonetic enhancement, that is, segmental lengthening/strengthening.

As a matter of fact, the direction of the morpho-phonetic effects are sometimes conflicting across studies and phenomena. For instance, in the same study by Rossi et al. (2026), the authors report that [stra] used as a prefix (as in It. *stratimido* “exceptionally shy”) is longer than in non-prefixed words (e.g., It. *strategico* “strategic”). Although this finding differs from the pattern they observe for [ino], it is consistent with previous evidence on pre-fixed words in other languages. For instance, Hay (2007) found that *un-* in New Zealand English is significantly longer in transparent forms such as *unburstable* than in opaque or lexicalized forms such as *unfortunate*. In the inflectional domain, while there are many studies showing that suffixal /s/ in English is shorter than non-suffixal /s/, a few have found the opposite effect (Seyfarth et al., 2017). One possible explanation focuses on the preponderance of the voiced allophone [z] in Seyfarth et al. (2017), and a pre-ponderance of the voiceless allophone [s] in Plag et al. (2017) and Schlechtweg and Corbett (2021, p. 386). Another possible interpretation relates to the different speech datasets on which the various studies have been conducted, i.e., either spontaneous speech corpora or laboratory elicited speech. In this respect, one may hypothesize that segments corresponding to morphemes are consistently enhanced in elicitation tasks as a side-effect of speakers' heightened attention to the formal properties of morphologically complex words, and particularly so when homophone or quasi-homophone pairs of words are used, as in the Seyfarth et al. (2017)'s study. Such task-related effects may override the production dynamics that typically emerge in natural speech, where the same segments may instead undergo shortening rather than lengthening.

Prior to this line of research, a widespread interpretation held that morpho-phonetic effects arise from the communicative enhancement (Jaeger and Buz, 2017) of “more meaningful” segments. According to this account, speakers exploit fine-grained details of acoustic implementation to protect morphological elements from reduction or cancellation (Hay, 2001, 2003), thereby enhancing the perceptibility of morphological properties. Within this framework, acoustic enhancement (typically manifesting as a durational increase) is construed as the conscious outcome of speaker hyperarticulation for the benefit of the listener. This explanation, however, has been challenged both by empirical findings reporting counterexamples and inconsistent relationships between morphological segmentability and acoustic duration, and by theoretical arguments questioning whether speakers can actively control such subtle phonetic distinctions (see e.g., Stein and Plag, 2022).

By contrast, another property that has been shown to influence the acoustic realization of segments in words is their paradigmatic probability. The degree of paradigmatic support that one forms receives from the lexicon predicts segment duration alongside phonological and contextual/syntagmatic factors: when segments are paradigmatically more probable (either because pertaining to smaller morphological families, or because supported by more morphologically similar words), they tend to be longer (e.g., Kuperman et al., 2007; Bell et al., 2021). For instance, Dutch plural /s/ is longer the more frequent the /s/ allomorph is compared to other plural allomorphs for a given noun, at least in those nouns where the plural is more frequent than the singular (Zee et al., 2021). Evidence of paradigmatic effects on duration also comes from languages with a richer morphology: in Estonian, for word-final inflection-marking /s/, smaller inflectional paradigms actively used by speakers (that is, with a greater paradigmatic probability) display longer durations (Lõo et al., 2023). For this kind of findings, a discriminative learning framework (Baayen et al., 2019, 2011) has proposed that phonetic effects reflect speakers' certainty about the functional contribution of a segment: segments tend to be phonetically enhanced when their functional role is highly consistent, and therefore predictable, and reduced when functional certainty is lower. For instance, Gahl and Baayen (2024) argued that durational variation in homophones reflects the strength of learned form-meaning mappings. Within the Discriminative Lexicon Model, words receiving stronger semantic support for their forms tend to exhibit longer durations, whereas weaker support is associated with phonetic reduction. In this view, frequency effects arise from learned discriminative associations rather than lexical frequency itself.

This perspective is consistent with the idea that sublexical elements may undergo phonetic enhancement when the association between form and meaning is particularly strong and certain, as is often the case for morphological endings encoding stable grammatical functions. Both the communicative function hypothesis and the semantic support hypothesis predict that morphological markers can undergo phonetic enhancement as a consequence of their reliable contribution to meaning. Unlike the former, however, the latter explicitly incorporates the opposing influence of lexical frequency (or lexical informativity), which favors reduction. Morphology-related phonetic patterns may therefore emerge from the interaction between enhancement pressures linked to form-meaning mappings and reduction pressures associated with lexical experience.

As is evident from this short outline, acoustic duration is most often targeted as a phonetic variable, but other speech production measures also support the same view. For instance, low vowels in inflected words (as in German *malt* “he paints”) are produced with a lower tongue body position than in non-inflected words (as in *kalt* “cold”), independently of word frequency and phonological factors (Saito et al., 2024). In Mandarin Chinese, fundamental frequency has been shown to vary to signal distinctions in the semantic space of words (e.g., Chuang et al., 2023; Lu et al., 2026). Such recent results broaden significantly the scope of research on the phonetic encoding of word meaning differences, considering that much of the existing literature has focused on the temporal domain when examining how phonetic detail contributes to the disambiguation of competing lexical forms and to morphological processing. The emphasis on duration is unlikely to be accidental, given that the micro-rhythm arising from durational relations between adjacent units of the speech signal – such as segments or syllables – has been argued to play a central role in the time-course of lexical access (e.g., Kemps et al., 2005).

In multi-segment (pseudo)affixes, phonetic effects are frequently found to vary across segments. For example, in a comparison between true-prefixed English words such as *distasteful* and *mistiming* and pseudo-affixed words like *distorted* and *mysterious*, Smith et al. (2012) found that morphological function increased the duration of the vowel /ɪ/ (and of /m/ in *mis-*), reduced the /s/, and had no effect on /d/. Similarly, Zee et al. (2025) observed that, in Dutch, the vowel in word-final /ən/ was longer, the nasal /n/ was shorter, and the degree of nasalisation decreased when the sequence functioned as a morphological affix (plural or infinitive), compared to when it did not. These results suggest that the co-presence of shortening and enhancement effects in the literature, besides reflecting different morphological properties and processing strategies, may also arise from the variability of the phonetic targets.

This also relates to the fact that morpho-phonetic effects do not target the morpheme only, but they tend to extend across different parts of the word. For instance, Hay (2003) showed that in a word like *exactly*, which is considerably more frequent than its base, *exact*, not only was the suffix -*ly* shorter, but the base itself more readily underwent /t/-deletion - an outcome less likely to be found in a word like *abstractly*, which is less frequent than its base, *abstract*. In the latter, -*ly* was longer and the /t/ in *abstract* tended to be preserved. Similarly, Seyfarth et al. (2017) investigated acoustic differences in English words bearing inflectional [z], [s], [t], or [d] (e.g., third person singular, noun plural, past tense, or past participle, as in *frees, laps, ducked*, and *tied*) compared to morphologically simple words ending in the same segments (e.g., *freeze, lapse, duct, tide*). They found that words with [s] or [z] as inflectional suffixes had significantly longer stems and suffixes than their morphologically simple homophones, although no such effect was observed for [t] and [d]. Such acoustic information is recoverable perceptually and used for lexical disambiguation: for instance, Dutch listeners are sensitive to the prosodic detail differentiating the stem of simplex singular nouns from their affixed plurals (Kemps et al., 2005). There are also more recent studies that clearly suggest that morpho-phonetic effects are global rather than local. Among them, the already mentioned study by Saito et al. (2024) reports that stem vowels in inflected words are produced with a lower tongue body position than in non-inflected words. Non-local acoustic effects further support the view that morphology-related phonetic variation is not merely a property of speech output, but reflects the operation of underlying representational and learning mechanisms. Patterns of phonetic enhancement and reduction may therefore provide indirect evidence about how form-meaning associations are encoded, accessed, and weighted during speech production.

This study contributes an additional piece of evidence by looking at production data for German /Cst/ word-final clusters, such as [pst] in *Obst* “fruit” (lexical or monomorphemic consonant cluster) as opposed to *tobst* “you rage” (morphological or bi-morphemic cluster, where /st/ is the second singular person marking of present indicative). Accordingly, the comparison necessarily involves simplex nouns and inflected verb forms, which constitute the relevant lexical contrast available in German for the phenomenon under investigation. In a previous exploratory study, we analyzed the duration of the whole consonant cluster, but we could not establish how much of that variation was due to the /st/ morpheme specifically, nor could we establish whether the preceding vowel was also involved in morpho-phonetic variation (Celata et al., 2022). Moreover, lexical frequency was controlled using a cumulative paradigm frequency measure, computed as the summed frequencies of all inflected forms belonging to the same lexeme. This choice effectively traded lexical-frequency variation for comparability across lexical classes and was motivated by the observation that simplex nouns and inflected verb forms differed dramatically in their individual word-form frequencies. However, by collapsing frequency across all inflected forms, this approach substantially reduced the lexical-frequency variation available to the statistical models. As a consequence, potential effects of individual word-form frequency were likely underestimated, and lexical frequency did not emerge as a significant predictor in any of the analyses.

In the current study, based on a slightly larger dataset and different acoustic measures, we extend the analysis in different directions. First, we want to assess whether all segments of the consonant cluster vary in the same way, or only the /st/ sequence, corresponding to the verbal morpheme in morphological clusters, changes. We also look at sub-segmental variation by analyzing the closure and aspiration phases of the final /t/, to verify how fine-grained morphologically conditioned variation can be. Second, we broaden our focus by looking at potential changes in the preceding vowel, corresponding to the stem's most sonorous element. By extending the analysis beyond the /st/ sequence, we investigate whether the observed effects are strictly local, i.e., limited to the segmental locus of the inflectional or derivational morpheme, or whether they also involve other portions of the word. Third, we include an analysis of non-durational properties – namely, intensity and center of gravity of two portions of aperiodic noise – in order to get a more detailed picture of the enhancement and/or reduction processes from the acoustic point of view. Fourth, we consider potential task-related variation, in light of the fact that the materials do not stem from a corpus of spontaneous speech, but from a controlled production task involving multiple repetitions of the same sentence materials. To that aim, we consider the different repetitions of the same items that the speakers produced as a source of potential acoustic variation. The hypothesis is that, under such controlled laboratory conditions, task-related factors may lead speakers to consciously hyperarticulate inflectional endings in target words with quasi-homophonous monomorphemic counterparts, resulting in lengthening rather in the shortening typically observed in production studies of less controlled speech. Fifth, we revise our treatment of lexical frequency. Unlike our previous exploration, which controlled lexical familiarity through cumulative paradigm frequency extracted from CELEX (Baayen et al., 1993), we model individual word-form frequency directly using frequency estimates from the more recent dlexDB lexical database (Kliegl et al., 2025), whose word-form counts provide a better match to the present study's focus on lexical variation across individual forms. In addition, the present analyses are based on a slightly expanded dataset, incorporating recordings from additional speakers who had been recorded as part of the original experiment but could not be included in the previous analyses because the manual segmentation required for the acoustic measurements had not yet been completed.

Taken together, these analyses allow us to evaluate whether morphology-related phonetic effects remain confined to the morphemic material itself or whether they interact with broader lexical and phonetic properties of the word. More generally, the present study asks whether morphologically conditioned phonetic variation is shaped solely by local morphological structure or whether it reflects probabilistic interactions with lexical and phonetic information during speech planning. Addressing this question is relevant to current models of speech production, regardless of the broader assumptions they make about the organization of linguistic representations.

## 2 Methods

### 2.1 Materials

16 German words (see Table 1) were selected, 8 of which contained a lexical cluster (such as *Wanst* “paunch”) and 8 a homophone morphological cluster (as in *bannst* “(you) banish,” *bann+st*). From now on the 2 groups of words will be referred to as LC and MC items respectively. The 2 groups were balanced for word length (calculated in number of syllables) and quality and quantity of the vowel preceding the cluster (with one exception for vowel quality). Word frequency was extracted from dlexDB (Kliegl et al., 2025). Figure 1 shows that LC items display higher word frequency and a broader frequency range compared to MC items, a pattern that plausibly stems from the lexical difference between the 2 sets (nouns vs. second-person singular verb forms). The 2 conditions correspond to different lexical categories (simplex nouns vs. inflected verb forms) reflecting the grammatical contrast through which the phenomenon is instantiated in German. The question addressed here is whether identical phonological material is realized differently when it forms part of the lexical representation of a simplex noun or functions as an exponent of grammatical information in an inflected verb form. Accordingly, lexical category is not treated as an alternative explanatory variable but as the grammatical environment in which these different functional roles are instantiated; there is no theoretical or empirical motivation to expect systematic phonetic differences between otherwise identical segmental sequences solely as a consequence of lexical category (Lohmann, 2020). Our hypothesis therefore concerns the linguistic function of the segmental material rather than lexical category itself.

**Table 1:** Experimental stimuli.

V	V length	Cluster	#C	Status	Item	Gloss
/a/	short	/nst/	three	LC	Wanst	paunch
				MC	bannst	banish-2SG
/ɛ/	short	/nst/	three	LC	Gespenst	ghost
				MC	verkennst	underestimate-2SG
/o:/	long	/pst/	three	LC	Obst	fruit
				MC	tobst	rave-2SG
/ʊ/	short	/lst/	three	LC	Wulst	bead
				MC	nullst	zero-2SG
/a/	short	/ŋkst/	four	LC	Angst	fear
				MC	langst	be enough-2SG
/ɛɒ/	long	/nst/	three	LC	Ernst	gravity
				MC	lernst	learn-2SG
/ɛɒ/	long	/pst/	three	LC	Herbst	Autumn
				MC	färbst	paint-2SG
/ɛ/, /ɔ/	short	/lpst/	four	LC	selbst	oneself
				MC	wölbst	bend-2SG

Diphthongs are considered “long vowels” because the annotation included both segments [ɛɒ] in the V interval. See Section 2.3 for further details.

**Figure 1:**
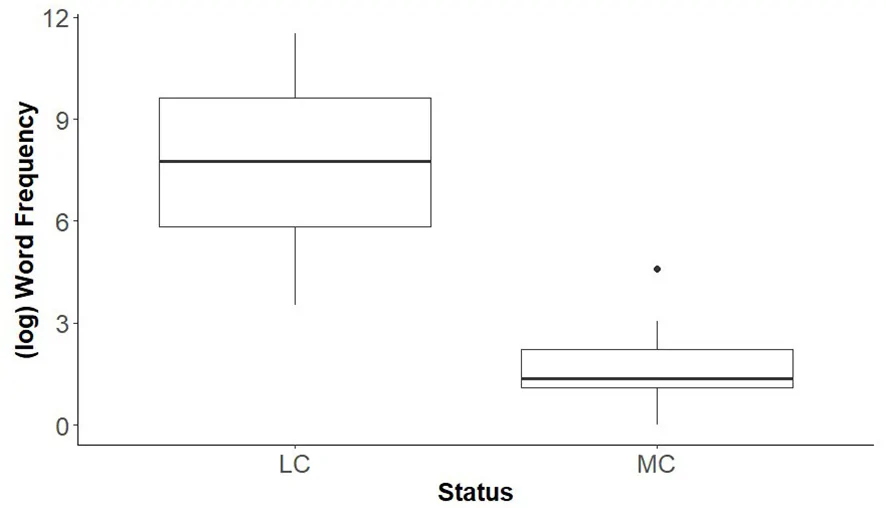
Log-word frequency by item class.

16 fillers (8 nouns and 8 verbs) were also selected. 10 were disyllables and 6 were monosyllables; they varied for word onset (2 of them beginning with a V, 9 with a C, 2 with a CC cluster, and 3 with a CCC cluster) and offset (5 ending with a V, 5 with a C, and 6 with a CC cluster).

### 2.2 Data elicitation procedure

For the reading task, each target word was included in 2 different carrier sentences, corresponding to 2 prosodic positions of the target word. In one case, the speakers had to read a question (*Was hast du gesagt?* “What did you say?”) followed by a reply in which the target word was in phrase-internal position (*Ich habe X gesagt* “I said X”). In the other case, both the question and the answer were slightly varied such that the target item was placed in phrase-final position (*Was sagst du heute? Heute sage ich X* “What do you say today? Today I say X”). It should be noted that the carrier sentences were neutral with respect to the meaning of the target words, which were consequently produced according to a “citation forms” speech style (with no preceding article or subject pronoun). We assume that, this way, coarticulatory and prosodic effects are controlled across items, while at the same time access to the meaning of the word is ensured by the pragmatic context evoked by the carrier sentence – unlike what might have occurred in the production of isolated words presented in list form.

15 native Standard Austrian German speakers (10 female, 5 male), aged 20–40, participated in the reading task. The speakers were recruited online via social media. We included only speakers who were born and raised in Vienna, had spent a large part of their life in Vienna, and had a university entrance diploma from secondary school (i.e., Matura). The recordings were conducted in a sound-attenuated booth at the Acoustics Research Institute of the Austrian Academy of Sciences in Vienna.

2 sentence lists had been created for the reading task. Speakers were divided into 2 groups (gender-mixed) according to the sentence list they were asked to read. Both sentence lists consisted of 48 different sentences and included all 16 filler sentences in both syntactic-prosodic conditions. More specifically, one list included 4 LC items and 4 non-corresponding MC items in both syntactic-prosodic conditions, and the other list included the other 4 LC and 4 MC items in both prosodic conditions. Each list was repeated twice so that each speaker read 96 sentences in total (each target item was repeated 4 times, for a total of 32 realizations of LC/MC clusters). The order of the sentences was randomized within each list, and we ensured that identical words in different prosodic conditions were not too close to each other (at least 10 different words intervening). Moreover, the order of the sentences was also randomized within each repetition, as to ensure that half of the items were in one prosodic condition and the other half in the other prosodic condition (and the reverse in the second repetition); this way, the 2 prosodic conditions were equally distributed not only across, but also within each repetition. The overall number of experimental items was 480 (15 speakers x 8 items x 2 prosodic conditions x 2 repetitions).

### 2.3 Analysis

The collected recordings were manually annotated in Praat (version 6.1.40; Boersma and Weenink, 2021). The annotation included orthographic transcriptions of the target words and segmentation of relevant vowels and consonants as explained below. All segmentation was carried out manually in Praat, with systematic cross-checking between waveform and spectrogram displays. Two illustrative examples are provided in Figure 2.

**Figure 2:**
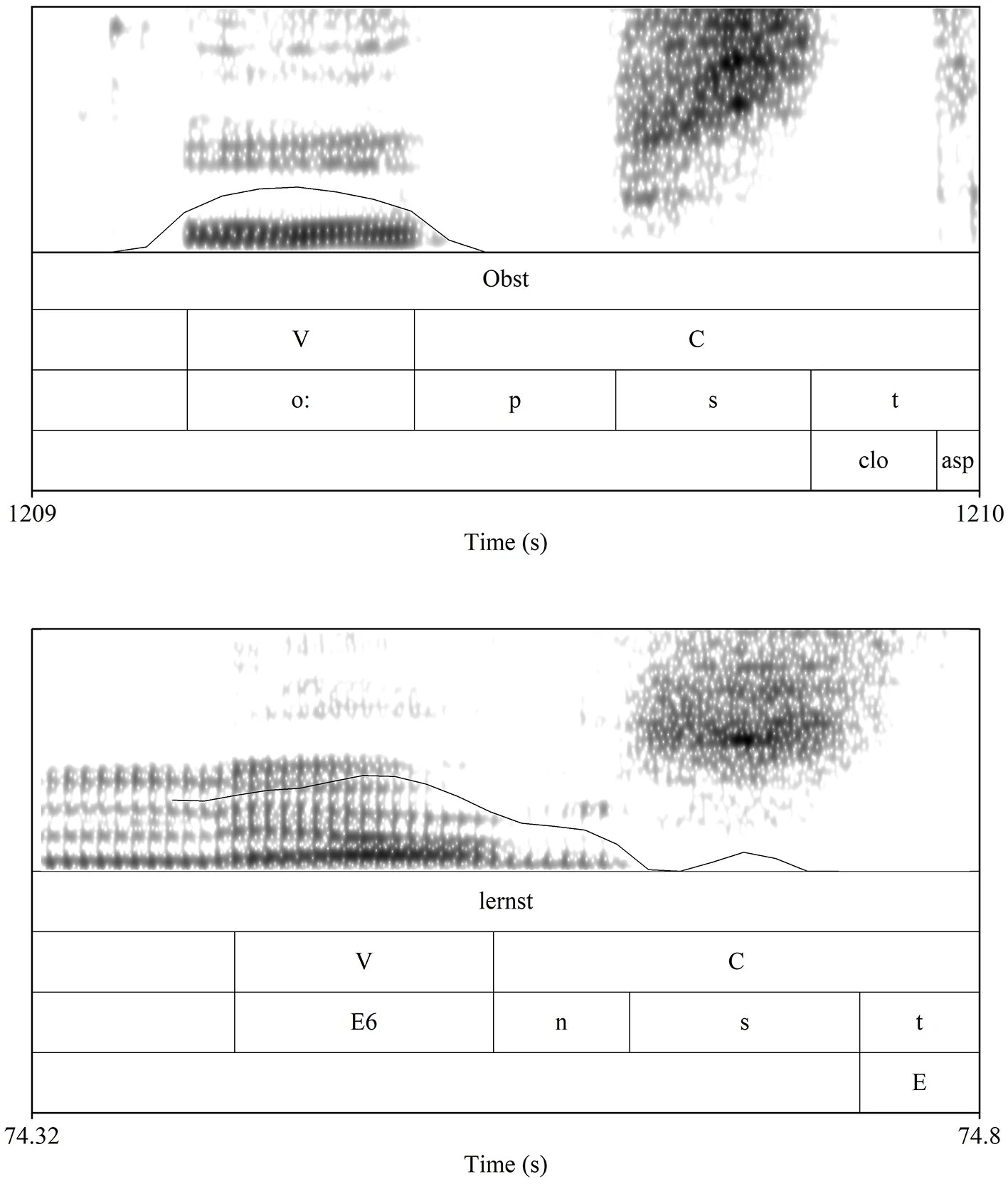
Examples of the segmentation for the word *Obst* (“fruit”, above) and the word *lernst* (“(you) learn”, below) including the spectrograms and intensity contours. The *lernst* example represents a case in which the /t/ closure and aspiration phases could not be reliably segmented and annotated.

Stimuli containing a rhotic were produced with a vocalized rhotic, as is standard in Austrian Standard German (Moosmüller et al., 2015). Accordingly, these items were annotated with a centralized diphthong ([ɛɒ] in IPA) in the V slot, followed by the consonant cluster; for example, in *Herbst*, V corresponded to [ɛɒ] and the consonantal interval to [pst].

Following established segmentation practices (e.g., Machac and Skarnitzl, 2009) and general descriptions of segmental reduction patterns in German (Kohler, 1990), segment boundaries were identified on the basis of amplitude minima, discontinuities in periodicity, formant transitions, and the presence of burst and aspiration noise. All boundaries were subsequently adjusted to the nearest positive zero-crossing in the waveform to ensure clean segmentation and to minimize measurement artifacts. The vowel-sonorant boundary (nasals and liquids) was determined by the presence of characteristic formant structures and a relatively stable amplitude plateau. The onset of /s/ following a stop was identified by a sudden increase in high-frequency frication (typically in the 3–8 kHz range), reflected in a sharp rise in spectral energy. However, when the stop exhibited a clearly visible burst, optionally followed by an aspiration phase, the onset of /s/ was placed immediately after the burst (or aspiration) offset.

Identifying the onset of /t/ following /s/ proved to be the most challenging segmentation task, due to the gradual decay of frication and the typically unmarked onset of oral closure in this context. When the onset of /t/ closure could not be clearly associated with an abrupt cessation of high-frequency frication and a concomitant drop in waveform amplitude, the intensity contour was consulted; in such cases, /t/ closure onset was marked at the point of visible intensity reduction. The release of /t/ was identified by a plosive burst, followed in most cases by aspiration, in line with previous descriptions of (Viennese) Austrian German as an aspirating variety (Moosmüller and Ringen, 2004) and with recent evidence indicating that younger speakers are increasingly lengthening the VOT of voiceless (fortis) stops as part of an ongoing sound change (Luef, 2020). However, in some tokens the internal boundary between the sibilant and the stop and/or the boundary between /t/ closure and /t/ aspiration could not be reliably identified. In these cases, the /st/ cluster was not internally segmented, and no acoustic measurements were extracted for either /s/ or /t/.

Segmentation was first performed by an expert phonetician and subsequently carried out independently by the first author. Initial inter-annotator discrepancies amounted to 32% of the total boundaries. These cases were then jointly reviewed by both annotators, revealing systematic sources of divergence related to (1) the identification of the /p/ burst vs. the onset of the following /s/; (2) the optional presence of low-intensity aperiodic noise between /l/ and /s/; and (3) the treatment of /t/ aspiration (inclusion vs. exclusion of the burst). Explicit decision criteria were subsequently established for these contexts, after which the first author revised all relevant segmentations.

Acoustic measurements were extracted using a Praat script and included the acoustic duration of:

- The whole consonant cluster;
- The C interval preceding the /st/ sequence;
- The /st/ sequence;
- The closure phase of the /t/;
- The aspiration phase of the /t/;
- The stressed vowel preceding the cluster.

Additionally, we extracted 4 measures not related to the temporal aspects of speech:

- The spectral center of gravity (COG) of /s/;
- The intensity of /s/;
- The spectral center of gravity (COG) of /t/ aspiration;
- The intensity of /t/ aspiration.

Average COG was extracted for the entire noise portion of each fricative and /t/ aspiration token with power set to “2” after applying a high-pass filter removing frequencies below 1,000 Hz. Measurements for intervals < 10 ms of duration were judged unreliable for COG analysis and discarded. Average intensity, in decibels (dB), was also extracted from the whole noisy portion. Intensity values were extracted in Praat, which calculates dB sound pressure levels based on root-mean-square (rms) amplitude over short temporal windows; the reported values correspond to the average intensity over the selected segment. To normalize for intensity differences across contexts, values were scaled by dividing them by the mean amplitude of the preceding vowel within the same word (e.g., Jongman et al., 2000).

While segmental duration has been widely investigated in the literature on morpho-phoentic variation, and intensity has been thoroughly studied as a correlate of acoustic enhancement or segmental reduction (e.g., Cangemi et al., 2018), our inclusion of the center of gravity (or first spectral moment) for the fricative and /t/ aspiration phase calls for further explanation. COG is commonly used as a measure of the average frequency of an aperiodic noise spectrum, weighted by its energy, and thus captures the distribution of spectral energy across the frequency domain (e.g., Gordon et al., 2002; Fuchs and Toda, 2010). Expressed in Hertz (Hz), COG values are frequently employed to investigate fricative place of articulation, with higher values typically associated with more anterior fricatives (e.g., /s/) and lower values with more posterior ones (e.g., /ʃ/; Jongman et al., 2000). Coarticulation also impacts on COG variation in fricatives, with labialization involved in both rounded vowels and labial consonants lowering the frequencies of the fricative noise (Koenig et al., 2013, and references therein). More relevant to the present study, however, COG has also been shown to correlate with the degree of consonantal reduction, with lower COG values indexing more reduced articulations (van Son and Pols, 1997; Zellers, 2017). Building on this work, the present study investigates whether COG variation can be observed as a function of the morphological status of the consonant cluster, once coarticulation effects have been controlled for. Specifically, lower /s/ COG values are expected to reflect consonantal reduction, whereas higher values would be indicative of acoustic enhancement.

For the /t/ aspiration interval, the interpretation of COG may differ, given the short duration and typically low intensity of this phase. In this context, the presence vs. absence of a plosive burst within the interval is expected to yield substantial differences in COG values, with the presence of a burst resulting in lower COG. Such effects are likely to override those associated with differences in constriction location. Consequently, higher COG values are expected to be associated with unreleased stops, which may be taken to reflect a greater degree of articulatory reduction.

Statistical analyses were conducted in R (R Core Team, 2024) using linear mixed-effects models. We used the lmerTest package (Kuznetsova et al., 2017) for model fitting, and the emmeans package (Lenth, 2022) for estimated marginal means and *post-hoc* pairwise comparisons.

Outliers were identified separately for each dependent variable using a ±2.5 SD criterion, computed within Speaker × Prosodic Position (internal vs. phrase-final). This procedure resulted in the removal of a very small proportion of observations (always below 1.5% for each variable). Observations could be excluded from one analysis but retained in others.

Overall, exclusions (including both outliers and missing data) were < 1% for cluster duration, C duration, V duration; ≈10% for /s/ intensity, /s/ COG, /t/ closure duration, /t/ aspiration duration, /t/ aspiration intensity; ≈14% for /t/ aspiration COG.

10 linear mixed-effects models were then fitted to test the influence of the experimental variables on each phonetic parameter. Fixed effects included: Status (MC vs. LC), Prosody (position within the carrier sentence: Internal vs. Final), Frequency (log-transformed token frequency), Repetition (1–4, indicating the different repetitions of an item by the same speaker), Vowel Length (short vs. long), Cluster Length (three vs. four segments). For COG analyses, a further predictor, Labiality, was included to account for clusters having a bilabial stop or a non-bilabial stop before /s/. Random intercepts for Speaker were included. Following the recommendations in Gahl and Baayen (2024) for datasets with very different lexical frequencies and item-bound predictors, random intercepts for Word were not fitted as lexical identity was intrinsically associated with the predictors of theoretical interest. In particular, each word occurred in only one Status condition and lexical frequency was explicitly modeled as a fixed effect. Following Gahl and Baayen (2024), we therefore treated the principal lexical sources of variation through theoretically motivated fixed predictors (Frequency, Vowel Length, Cluster Length, and Labiality where appropriate), rather than by including an additional Word random intercept. Each model was initially set up to include all fixed effects and two-way interactions of Status with each of the other predictors.

Afterwards, we performed stepwise model selection using the step() function in the lmerTest packages to assess whether Status was retained as a main effect and/or as a part of a two-way interaction. The stepwise procedure applied backward elimination based on Type III F-tests (Satterthwaite approximation), iteratively removing non-significant terms. Significance threshold was set at *p* < 0.001. This approach was adopted to identify the most parsimonious models for each dependent variable while limiting unnecessary model complexity relative to the size of the lexical dataset and the number of candidate predictors.

The resulting models in which Status was included were further analyzed; those in which Status was removed through the stepwise selection due to non-significance were discarded, as such outcome indicated that the other control variables, rather the morphological Status, accounted for the observed variation of the acoustic feature. Model adequacy was assessed by means of residual diagnostics, including Q–Q plots and residuals-vs.-fitted plots. In most cases, visual inspection indicated an adequate approximation to normality, with good alignment across the central quantiles, only minor deviations in the tails, and no evidence of heteroscedasticity or other systematic structure. For those cases in which the inspection revealed a less regular residual distribution, the point is commented in the Results section. All model outputs and Q–Q plots are provided in the Appendix.

## 3 Results

Of the 10 models implemented, 5 retained Status as a significant predictor. 3 of these involved durational variables, namely cluster duration, vowel duration, and /st/ duration, while 2 involved spectral variables, specifically /s/ intensity and /s/ COG.

In the following section, we examine in detail the effect of Status and other predictors within each relevant model, starting with the durational variables. Main effects are discussed only when they were not included in interactions; otherwise, interpretation relies on LC–MC contrasts derived from estimated marginal means. Tables therefore summarize the relevant contrasts, and only effects reaching the predefined significance threshold (*p* < 0.001) are interpreted.

### 3.1 Effects on duration

In all models involving durational variables, a robust main effect of Prosody was observed, with segments produced in phrase-final position being substantially longer than those produced in phrase-internal position. Estimated differences are reported in the models listed in Appendix 1. As Prosody did not enter into any interaction, this effect can be interpreted as global and is therefore not discussed further. Repetition did not emerge as a significant predictor in any model.

#### 3.1.1 Cluster duration

A significant Status × Frequency interaction (Table 2) indicates that the durational contrast between LC and MC clusters varies as a function of lexical frequency. Simple contrasts for Status show that MC clusters are significantly shorter than LC clusters at low frequency values (e.g., Δ ≈ –56 ms at logFreq = –2; Δ ≈ –47 ms at logFreq = –1; all *p* < 0.001), with the magnitude of the LC–MC contrast progressively decreasing as frequency increases and becoming non-significant at higher frequency values. Importantly, simple contrasts for Frequency (not shown in the table) reveal that frequency-driven reduction is attested only in LC items, where cluster duration decreases monotonically as frequency increases, whereas no frequency-related modulation is observed in MC items, for which none of the consecutive frequency contrasts reaches significance. This asymmetry suggests that frequency-based temporal reduction primarily targeted LC clusters, while MC clusters appeared relatively resistant to frequency-related durational modulation.

**Table 2:** LC-MC contrasts for cluster duration.

Conditioning factor	Level	MC – LC (ms)	SE	df	*t*	*p*
**Frequency**	logFreq = −2	**−56.4**	15.0	456	−3.76	<**0.001**
	logfreq = −1	**−47.4**	13.4	456	−3.54	<**0.001**
	logfreq = 0	−38.3	12.2	455	−3.15	0.002
	logfreq = 1	−29.3	11.5	455	−2.55	0.011
	logfreq = 2	−20.3	11.5	454	−1.76	0.078
	logfreq = 3	−11.2	12.1	454	−0.93	0.354
**Vowel length**	short	−8.5	9.8	454	−0.87	0.384
	long	**−67.9**	16.3	456	−4.17	<**0.001**
**Cluster length**	three	−10.2	8.4	454	−1.22	0.225
	four	**−66.2**	17.8	456	−3.73	<**0.001**

Negative values indicate shorter MC clusters relative to LC clusters. Estimates are based on estimated marginal means derived from the fitted mixed-effects model. Boldface indicates contrasts significant at *p* < 0.001.

The Status × Vowel Length interaction further qualifies the LC–MC contrast. In short-vowel contexts, no reliable durational difference is observed between MC and LC clusters (Δ ≈ –9 ms, n.s.). In contrast, in long-vowel contexts, MC clusters are substantially shorter than LC clusters (Δ ≈ –68 ms, p < 0.001). Thus, the effect of morphological status on cluster duration emerged selectively in long-vowel words.

A further interaction between Status and Cluster Length shows that the LC–MC contrast is modulated by consonantal complexity. For three-consonant clusters, no significant durational difference emerges between MC and LC clusters (Δ ≈ –10 ms, n.s.). By contrast, for four-consonant clusters, MC clusters are again significantly shorter than LC clusters (Δ ≈ –66 ms, *p* < 0.001). This indicates that the durational correlates of morphological status were expressed primarily in words with more complex consonantal configurations.

#### 3.1.2 Vowel duration

In addition to the main effect of Prosody, a strong main effect of Vowel Length confirms that phonologically long vowels and diphthongs are substantially longer than short vowels (Δ ≈ +44 ms), independent of Status. The effect of Vowel Length did not interact with Status and is therefore interpreted as global.

A significant Status × Frequency interaction (Table 3) indicates that the durational contrast between LC and MC vowels varies as a function of lexical frequency. Simple contrasts for Status show that no reliable difference between MC and LC vowels is observed at low frequency values, whereas MC vowels become progressively longer than LC vowels as frequency increases. Specifically, the LC–MC contrast becomes significant at mid frequency values (Δ ≈ +12 ms at logFreq = 0, *p* = 0.008) and increases steadily at higher frequencies (Δ ≈ +19 ms at logFreq = 1; Δ ≈+26 ms at logFreq = 2; Δ ≈ +32 ms at logFreq = 3; all *p* < 0.001). Importantly, simple contrasts for Frequency reveal opposite frequency effects across Status conditions: vowel duration decreases with increasing frequency in LC items, whereas it increases with increasing frequency in MC items, with all consecutive frequency contrasts being significant within each Status condition. This crossover pattern indicates that frequency-related temporal modulation affects LC and MC vowels in systematically different ways.

**Table 3:** LC-MC contrasts for vowel duration.

Conditioning factor	Level	MC – LC (ms)	SE	df	*t*	*p*
**Frequency**	logFreq = −2	−2.0	3.9	454	−0.50	0.614
	logFreq = −1	4.9	4.0	454	1.23	0.219
	logFreq = 0	11.8	4.5	454	2.65	0.008
	logFreq = 1	**18.7**	5.2	454	3.61	<**0.001**
	logFreq = 2	**25.5**	6.1	454	4.21	<**0.001**
	logFreq = 3	**32.4**	7.1	454	4.60	<**0.001**

Contrasts are expressed as MC - LC (positive values indicate longer vowels in MC items). Estimates are based on emmeans derived from the fitted mixed-effects model. Boldface indicates contrasts significant at *p* < 0.001.

#### 3.1.3 /st/ duration

As already highlighted, in MC items the /st/ sequence corresponds to the inflectional morpheme. Overall, the results of this model show a clear convergence with those obtained for the larger unit, namely cluster duration, pointing to a consistent pattern across levels of analysis.

A significant Status × Frequency interaction indicates that the LC–MC contrast in /st/ duration is modulated by lexical frequency. Simple contrasts (Table 4) show that MC /st/ sequences are substantially shorter than LC sequences at low frequency values (e.g., Δ ≈ –49 ms at logFreq = –2; Δ ≈ –40 ms at logFreq = –1; *p* < 0.001). The magnitude of the LC–MC contrast decreases monotonically as frequency increases and becomes non-significant at higher frequency values. This pattern indicates that frequency-driven temporal reduction primarily affects LC clusters, whereas MC clusters are comparatively resistant to frequency-related shortening.

**Table 4:** LC-MC contrasts for /st/ duration.

Conditioning factor	Level	MC – LC (ms)	SE	df	*t*	*p*
**Frequency**	logFreq = −2	**–48.5**	11.1	455	−4.37	<**0.001**
	logFreq = −1	**–39.6**	9.9	455	−4.01	<**0.001**
	logFreq = 0	−30.8	9.0	455	−3.42	0.001
	logFreq = 1	−21.9	8.5	454	−2.58	0.010
	logFreq = 2	−13.0	8.4	453	−1.54	0.124
	logFreq = 3	−4.2	8.9	453	−0.47	0.641
**Vowel length**	short	−10.8	7.2	453	−1.50	0.133
	long	**–50.5**	12.0	455	−4.20	<**0.001**
**Cluster length**	three	−9.4	6.2	453	−1.52	0.129
	four	**–51.9**	13.1	455	−3.96	<**0.001**

Contrasts are expressed as MC - LC (negative values indicate shorter /st/ duration in MC items). Boldface indicates contrasts significant at *p* < 0.001.

The Status × Vowel Length interaction further qualifies the LC–MC contrast. In short-vowel contexts, no reliable durational difference is observed between MC and LC clusters. In long-vowel contexts, MC clusters are significantly shorter than LC clusters (Δ ≈ –51 ms, *p* < 0.001), indicating that the effect of morphological status on /st/ duration emerges selectively in long-vowel items.

A further interaction between Status and Cluster Length shows that the LC–MC contrast is conditioned by consonantal complexity. No reliable difference between MC and LC clusters is observed for three-consonant clusters, whereas in four-consonant clusters MC clusters are again substantially shorter than LC clusters (Δ ≈ –52 ms, *p* < 0.001). This suggests that morphological status affects /st/ duration primarily in structurally more complex cluster configurations.

Visual inspection of the Q-Q plot of residuals shows a generally good alignment with the theoretical normal distribution in the central quantiles, indicating that the model captures the bulk of the variance adequately. Some deviation is visible in the upper tail, with a small number of positive residuals exceeding the reference line. This pattern suggests mild right-tail heaviness.

### 3.2 Effects on spectral properties

#### 3.2.1 /s/ intensity

Given the interaction between Status and Frequency, interpretation focuses on LC–MC contrasts (expressed as MC - LC) derived from estimated marginal means (Table 5). Simple contrasts for Status show that MC items exhibit higher /s/ intensity than LC items at the lowest frequency level (Δ ≈ +0.065 at logFreq = –2), with the LC–MC contrast reaching the predefined significance threshold (*p* < 0.001). At higher frequency levels, the magnitude of the LC–MC contrast progressively decreases and becomes non-significant. Simple contrasts for Frequency further reveal an asymmetric pattern across Status conditions. While LC items show a systematic increase in /s/ intensity as frequency increases (all consecutive frequency contrasts significant at *p* < 0.001), MC items do not exhibit reliable frequency-driven modulation. Again, this indicates that frequency-related intensity enhancement primarily affects LC items rather than MC items.

**Table 5:** LC-MC contrasts for /s/ intensity.

Conditioning factor	Level	MC – LC (ms)	SE	df	*t*	*p*
**Frequency**	logFreq = −2	**0.065**	0.018	414	3.61	**0.0003**
	logFreq = −1	0.050	0.016	414	3.09	0.0022
	logFreq = 0	0.035	0.015	413	2.34	0.0196
	logFreq = 1	0.019	0.014	413	1.37	0.1711
	logFreq = 2	0.004	0.014	412	0.26	0.7919
	logFreq = 3	−0.012	0.015	412	−0.81	0.4190
**Vowel length**	short	0.014	0.012	412	1.21	0.2278
	long	0.057	0.020	414	2.86	0.0044
**Cluster length**	three	0.002	0.010	413	0.19	0.8467
	four	0.069	0.022	414	3.19	0.0015

Contrasts are expressed as MC - LC (positive values indicate higher /s/ intensity in MC items). Estimates are based on emmeans averaged over the remaining predictors. Contrasts meeting the predefined significance threshold (*p* < 0.001) are reported in bold.

The model also outputs a significant interaction of Status with Vowel Length and an interaction of Status and Cluster Length. As for the first one, no reliable LC–MC contrast emerges in short-vowel contexts and for three-consonantal clusters; in long-vowel contexts and in four-consonant clusters, MC items show numerically higher /s/ intensity than LC items (Δ ≈ +0.057 and +0.069, respectively), but the contrasts do not meet the predefined significance threshold and are therefore not interpreted as reliable.

#### 3.2.2 /s/ center of gravity (COG)

A robust main effect of Prosody was observed, with /s/ COG being significantly lower in phrase-final than in phrase-internal position (Δ ≈ –317 Hz, *p* < 0.001). As Prosody does not enter into any interaction with Status, this effect is interpreted as global.

A strong Status × Frequency interaction indicates that LC and MC items differ systematically in how /s/ COG varies with lexical frequency. Simple contrasts for Frequency (Table 6) show that both LC and MC items exhibit increasing COG values as frequency increases, but the effect is markedly stronger for MC items. Simple contrasts for Status reveal that MC items have significantly higher /s/ COG than LC items across almost the entire frequency range, with the LC–MC contrast increasing monotonically from low to high frequency values (from Δ ≈ +1,500 Hz at logFreq = –1 to Δ ≈ +3,500 Hz at logFreq = 3; all *p* < 0.001). This pattern indicates that frequency-related spectral enhancement is substantially amplified in MC items.

**Table 6:** LC-MC contrasts for /s/ center of gravity.

Conditioning factor	Level	MC – LC (ms)	SE	df	*t*	*p*
**Frequency**	logFreq = −2	1,018	315	416	3.24	0.0013
	logFreq = −1	**1,507**	295	415	5.12	<**0.0001**
	logFreq = 0	**1,996**	302	414	6.61	<**0.0001**
	logFreq = 1	**2,486**	336	413	7.41	<**0.0001**
	logFreq = 2	**2,975**	388	412	7.66	<**0.0001**
	logFreq = 3	**3,464**	454	411	7.64	<**0.0001**
**Vowel length**	short	**2,911**	370	412	7.87	<**0.0001**
	long	1,015	331	415	3.07	0.0023
**Cluster length**	three	**2,420**	306	412	7.91	<**0.0001**
	four	**1,506**	367	415	4.11	<**0.0001**
**Labiality**	no	**914**	226	414	4.04	<**0.0001**
	yes	**3,012**	418	413	7.21	<**0.0001**

Contrasts are expressed as MC - LC (positive values indicate higher /s/ COG in MC items). Contrasts meeting the predefined significance threshold (*p* < 0.001) are reported in bold. Estimates are based on emmeans averaged over the remaining predictors.

The LC–MC contrast is also modulated by Vowel Length. In short-vowel contexts, MC items show substantially higher /s/ COG than LC items (Δ ≈ +2,910 Hz, *p* < 0.001). A similar but weaker difference is observed in long-vowel contexts (Δ ≈ +1,015 Hz), which is however non significant.

A further interaction between Status and Cluster Length indicates that the LC–MC contrast is robust across levels of consonantal complexity. MC items exhibit significantly higher /s/ COG than LC items in both three- and four-consonant clusters (Δ ≈ +2,420 Hz and Δ ≈ +1,506 Hz, respectively; *p* < 0.001), with a larger contrast in less complex clusters.

Finally, the Status × Labiality interaction reveals that the LC–MC contrast in /s/ COG is present in both non-labial and labial contexts, but is markedly stronger when Labiality is present (Table 6). While MC items show higher COG values than LC items in non-labial environments (Δ ≈ +914 Hz, *p* < 0.001), the contrast is larger in labial contexts (Δ ≈ +3,010 Hz, *p* < 0.001). This indicates that labial coarticulation amplifies the spectral differentiation associated with morphological status.

## 4 Discussion

The present study set out to investigate the phonetic correlates of morphological status in German word-final /Cst/ clusters by jointly examining temporal and non-temporal acoustic parameters across multiple domains. By adopting a multi-parameter approach and systematically modeling interactions between morphological status and other linguistic factors which constrain variability across the target items, the results provide a nuanced picture of how morpho-phonetic effects are distributed across segments, acoustic dimensions, and lexical subsets characterizing the experimental dataset.

In our data, morphological complexity was associated with widespread durational reduction, affecting not only the morphemic material but also neighboring phonetic segments, consistent with accounts positing distributed processing mechanisms. More generally, the present findings contribute to a growing body of evidence that phonetic implementation is sensitive to interactions between morphological, lexical, and phonological information, a pattern that is compatible with models that assume less strictly modular processing. The observed interactions between morphological structure, lexical properties, and phonetic realization suggest that information traditionally assigned to different levels of linguistic processing remains available throughout speech production. As such, morphology-related phonetic variation offers a valuable behavioral window onto the mechanisms through which learned form-meaning associations shape speech (Gahl and Baayen, 2024; Hay, 2003; Pierrehumbert, 2002; Stein and Plag, 2021).

A first general observation concerns the conditional nature of Status-related effects. Across all models, morphological status never emerged as main effect; instead, its phonetic correlates consistently surfaced through interactions with several other predictors, most notably lexical frequency, vowel length, and cluster complexity. This pattern suggests that, in our dataset, morpho-phonetic effects are context-sensitive phenomena whose realization depends on the interaction between morphology and other lexical or phonological dimensions. In other words, once phonological and lexical determinants of segmental duration have been accounted for, a residual component of phonetic variation remains that reflects the functional role of the segments within the word. In the present case, identical phonetic material is realized differently depending on whether it contributes to the grammatical identification of an inflected verb form or merely constitutes part of the lexical representation of a simplex noun.

At the same time, the effects observed are far from weak or incidental. They are replicated across several dependent variables and often show converging directions across levels of analysis, lending robustness to the overall pattern. Given the limited size of the stimulus set, the present results should be interpreted as demonstrating how these interactions operate within the lexical space sampled here. Establishing their quantitative generality will require larger datasets, where frequency and phonological structure can be dissociated more reliably from item-specific idiosyncrasies, consistently with recommendations from mixed-effects modeling approaches (Westfall et al., 2014).

A result that cuts across all models concerns Prosody. Although prosodic position systematically affects several acoustic parameters, it never interacts with morphological status. This dissociation suggests that prosodic strengthening and morpho-phonetic modulation operate as independent dimensions of phonetic organization. In other words, prosodic effects appear to be applied globally and uniformly, without selectively enhancing or attenuating the phonetic correlates of morphological structure. This separation supports a view in which prosody provides a stable scaffolding for phonetic realization, while morphological effects modulate phonetic detail within that framework.

A similar conclusion can be drawn for Repetition. Across all models, Repetition neither emerges as a significant predictor, nor interacts with Status. This indicates that the observed morpho-phonetic patterns are not driven by task-related repetition effects, such as practice, fatigue, or short-term adaptation. Rather, the stability of Status-related effects across repetitions supports their interpretation as structural properties of the lexical items under investigation, rather than artifacts of the experimental procedure.

Turning to the temporal domain, a consistent pattern emerges across models involving cluster duration, /st/ duration, and vowel duration. While the /st/ sequence corresponds to the inflectional morpheme in MC items, the effect of Status is not confined to this local domain. Instead, temporal modulation extends to the entire consonant cluster and, in the opposite direction, to the preceding vowel. This provides clear evidence that morpho-phonetic effects in the present data are global rather than local, affecting larger prosodic or rhythmic units rather than being restricted to the segment that directly encodes morphological information, consistently with previous evidence in the literature (e.g., Saito et al., 2024; Seyfarth et al., 2017).

Where temporal effects of Status do emerge, they are consistently reduction effects, in agreement with recent evidence, issued by lab speech and corpus data, on inflectional /s/ variations in English (Plag et al., 2017, 2020; Schmitz et al., 2021) and German (Schmitz and Baer-Henney, 2024) as well as different morphemes and languages (Seifart, 2025; Rossi et al., 2026), and contrary to lab-speech studies based on (quasi-)homophone word elicitation (e.g., Seyfarth et al., 2017; Smith et al., 2012) and prefix production (e.g., Hay, 2007; Rossi et al., 2026), reporting durational increase. In several models, MC items exhibit shorter cluster or /st/ durations than their LC counterparts, particularly in words characterized by low frequency values and in structurally complex configurations. Rather unexpectedly since we used lab-speech and a homophone elicitation paradigm, this pattern aligns with studies reporting shortening of inflectional material in spontaneous speech and stands in contrast to a strong version of listener-oriented hyperarticulation accounts, which would predict systematic lengthening of morphologically informative segments.

The temporal reduction observed on clusters and on the /st/ sequence is accompanied by an opposite effect on the preceding vowel. In MC items, vowels become progressively longer than in LC items as frequency increases, yielding a clear crossover pattern. This opposing behavior mirrors patterns reported for other languages (Zee et al., 2025; Smith et al., 2012) and points to a process of rhythmic compensation rather than uniform temporal reduction. If confirmed, such a pattern would suggest that morphological status does not simply trigger temporal shortening, but instead reorganizes the temporal structure of the word as a whole, potentially involving lengthening or acoustic enhancement of selected segments (cf. a similar argument in Seifart, 2025). This outcome would be expected also under the discriminative learning approach, that has shown that the duration effects can be conceptualized as emerging in language processing from the relations of form and meaning in the lexicon (Baayen et al., 2019; Gahl and Baayen, 2024).

Beyond duration, the study provides clear evidence that morphological status also affects non-temporal acoustic parameters, specifically the intensity and spectral center of gravity of /s/. In a subset of MC items, /s/ intensity is higher than in corresponding LC items, particularly at low frequency values. This result is counterintuitive if morphological status is assumed to induce a general reduction process. Instead, it suggests that temporal reduction may be partially compensated by increased energy, pointing to a more complex redistribution of acoustic cues rather than uniform weakening.

An even more striking result concerns the center of gravity of /s/. Contrary to expectations based on previous work linking lower COG values to consonantal reduction, MC items consistently exhibit higher COG values than LC items, with the LC–MC contrast increasing monotonically with frequency. Higher COG values are typically associated with more focused fricative noise, higher-frequency energy concentration, and greater articulatory precision, yielding a more canonical realization of /s/. This finding suggests that, in MC items, temporal reduction is not accompanied by spectral weakening, but rather by spectral sharpening. In other words, morphological status in our dataset appears to trigger reduced duration but increased articulatory precision, highlighting the limits of models that focus exclusively on duration and implicitly conflate shortening with acoustic reduction. Importantly, the COG effect persists after controlling for Labiality, ruling out an explanation in terms of coarticulation with adjacent consonants. This strengthens the interpretation of the COG increase as a controlled phonetic adjustment rather than a by-product of segmental context.

By contrast, no effects of Status emerge for subsegmental intervals, such as /t/ closure or /t/ aspiration. This indicates that morpho-phonetic effects in the present data do not penetrate the finest-grained temporal domains, suggesting that morphological information is not encoded at the level of micro-articulatory timing, but rather at higher-level segmental or cluster-based units.

Lexical frequency emerged as a robust predictor across virtually all analyses. This finding supports the revised treatment of lexical frequency adopted in the present study, where we modeled individual word-form frequency directly, rather than controlling lexical familiarity through cumulative paradigm frequency (Celata et al., 2022). In particular, 3 out of 5 models reveal frequency-related effects that are more robustly attested in LC than in MC items. Specifically, models of cluster duration, /st/ duration and /s/ intensity show that frequency-driven reduction emerges only in LC items, where consonant duration decreases and intensity increases monotonically with increasing frequency, whereas no comparable frequency-related modulation is observed in MC items. Only in the /s/ COG model, frequency effects were stronger in MC items; in the Vowel duration model, both classes were affected by frequency in the same way. This asymmetric pattern must be interpreted with caution. As highlighted in the description of the materials, LC items span a substantially wider frequency range and show greater dispersion than MC items, which are on average less frequent and clustered within a narrower portion of the frequency spectrum. As a consequence, the asymmetry in the results possibly reflects the broader frequency range characterizing LC items.

Frequency-driven modulation observed in cluster and /st/ durations is restricted to LC items, a pattern consistent with recent evidence showing that frequency effects are modulated by morphological complexity and are more readily detected in morphologically simple than in polymorphemic words (e.g., Saito et al., 2024). By contrast, in other cases – most notably vowel duration – frequency affects both LC and MC items, albeit in opposite directions: while LC items exhibit the expected shortening with increasing frequency, MC items show vowel lengthening. This apparent paradox suggests that frequency may operate through different dynamics in the vocalic and consonantal domains. Vowel lengthening in high-frequency MC items could be interpreted, within a paradigmatic probability framework (e.g., Kuperman et al., 2007; Gahl and Baayen, 2024; Saito et al., 2024), as reflecting greater support for stem vowels in high-frequency inflected verbal forms compared to corresponding vowels in high-frequency singular noun forms. In contrast, consonantal effects appear more compatible with canonical frequency-driven reduction in monomorphemic words, yielding shortening in high-frequency LC items and no comparable effect in MC items. In the current dataset, frequency apparently operates through different dynamics in the vocalic and consonantal domains. The vowel lengthening observed in high-frequency MC items could reflect stronger support for forms whose morphological contribution is highly reliable and consistently associated with a specific grammatical meaning, partially counteracting the reduction pressure typically associated with frequency. This is reminiscent of recent discriminative learning accounts of phonetic variation arguing that frequency-related effects cannot be reduced to lexical frequency alone but emerge from the interaction between frequency and the strength of learned form-meaning mappings (Gahl and Baayen, 2024). An alternative explanation is that the consonantal cluster in MC items, being inherently shorter as a consequence of its morphological status, may be subject to a threshold effect that limits the applicability of further frequency-related reduction.

As already noted, any of these hypotheses would require a larger and more diverse dataset to be systematically tested, disentangling genuine frequency effects from item-specific and distributional constraints. The present findings nonetheless suggest that frequency effects in MC items cannot be straightforwardly interpreted as instances of canonical frequency-induced reduction, but rather reflect complex interactions between frequency, morphological structure, and word-level temporal organization. A corpus study would provide an important extension of the present work by allowing these interactions to be examined across a broader range of lexical items and distributional environments than is possible in a controlled laboratory design.

In conclusion, the results suggest that morpho-phonetic effects in German /Cst/ clusters are neither purely restricted to the morphemic strings that may trigger them, nor purely durational. Instead, they involve a complex redistribution of temporal and spectral cues, which varies across lexical subsets and is sensitive to lexical characteristics such as frequency and phonological structure. Morphological status thus shapes not only how long segments are but also how they are realized acoustically, pointing to a complex interaction between morphology and phonetic detail. The context-dependent nature of the observed effects is difficult to reconcile with accounts assuming encapsulated linguistic modules, but is compatible with models in which phonetic realization emerges from the interaction of multiple probabilistic sources of information. More broadly, the present results provide empirical evidence that these sources of information jointly contribute to shaping phonetic realization during speech planning, thereby informing current models of how complex linguistic information is coordinated in language production.

## Data Availability

The original contributions presented in the study are included in the article/Supplementary material, further inquiries can be directed to the corresponding author.
