# Supplementary material for: Morphology beyond the morpheme: distributed processing effects in the acoustic realization of German word-final clusters
Source: Front Hum Neurosci. 2026 Oct 6;20:1924343. doi: 10.3389/fnhum.2026.1924343 (PMC13680481; doi:10.3389/fnhum.2026.1924343)
Supplement: Supplementary file 1 [file Data_Sheet_1.pdf]

Supplementary Material

1 APPENDIX

Model outputs and Q-Q plots for the models in which Status was retained after stepwise selection.

1.1 Linear mixed-effects model for cluster duration (Cldur)

Fixed effect	Estimate	SE	df	t value	p value
(Intercept)	249.93	10.50	18.05	23.81	$<$ .001
Status (MC)	19.38	8.51	454.21	2.28	.023
Prosody (final)	90.05	3.34	454.01	26.93	$<$ .001
log.WordFreq	-9.97	1.99	455.88	-5.01	$<$ .001
VowelLength (long)	20.37	6.57	455.57	3.30	.001
ClusterLength (four)	76.89	11.38	456.30	6.76	$<$ .001
Status $\times$ log.WordFreq	9.03	2.18	455.18	4.14	$<$ .001
Status $\times$ VowelLength	-59.39	11.70	455.44	-5.08	$<$ .001
Status $\times$ ClusterLength	-56.00	13.40	455.68	-4.18	$<$ .001
Random effects					
Group	Variance		SD		
Speaker (Intercept)	1413		37.59		
Residual	1333		36.51		

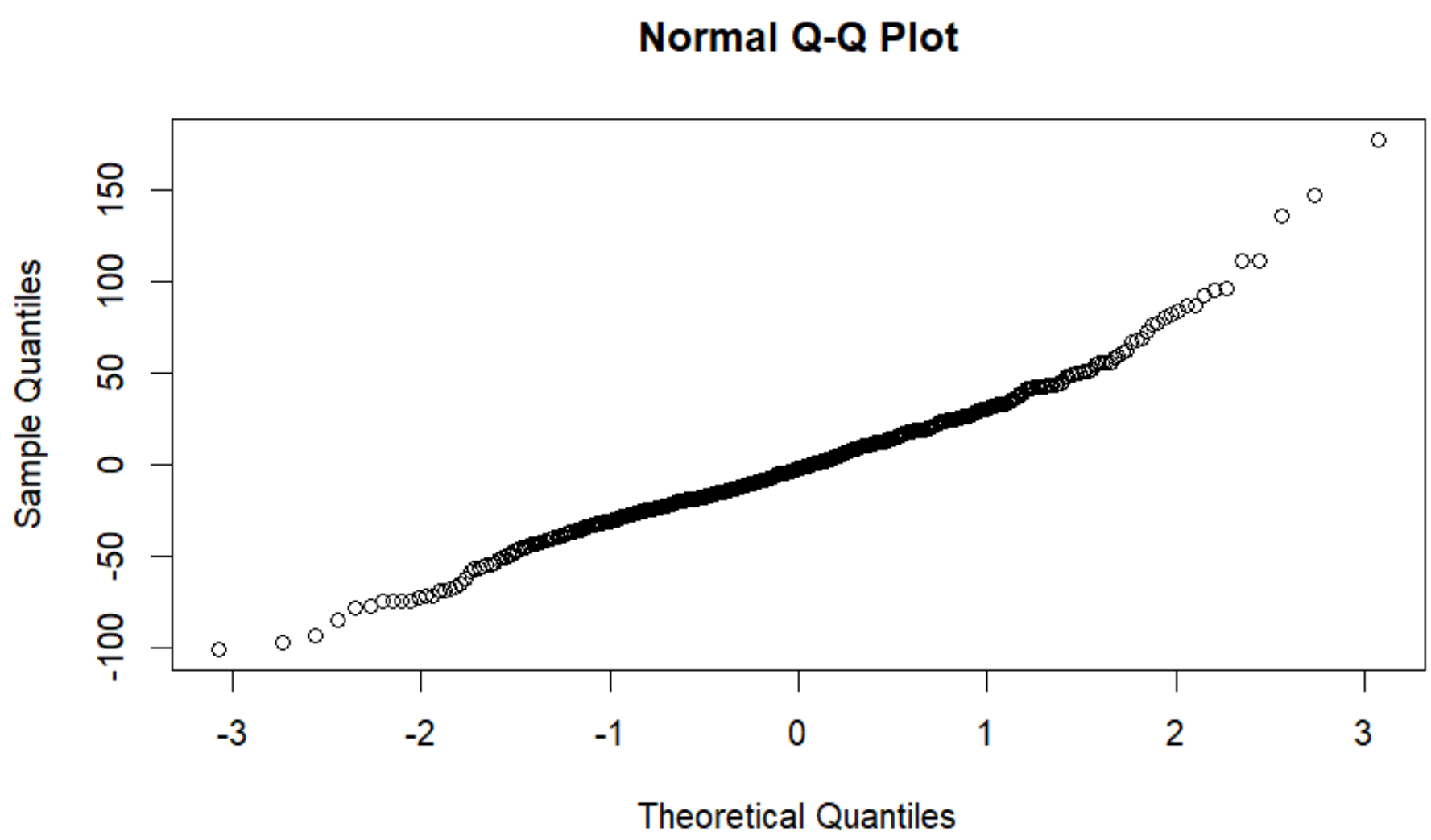

Figure S1. Q-Q plot of residuals for the cluster duration model.

1.2 Linear mixed-effects model for vowel duration (Vdur)

Fixed effect	Estimate	SE	df	t value	p value
(Intercept)	95.77	5.56	24.84	17.23	$<$ .001
Status (MC)	11.79	4.46	454.13	2.65	.008
Prosody (final)	10.68	2.16	454.00	4.95	$<$ .001
log.WordFreq	-1.93	0.59	454.36	-3.26	.001
Repetition 2	-0.01	3.05	454.00	-0.00	.998
Repetition 3	-7.93	2.96	454.03	-2.68	.008
Repetition 4	-4.36	3.00	454.03	-1.45	.147
VowelLength (long)	43.84	2.44	454.60	17.99	$<$ .001
Status $\times$ log.WordFreq	6.87	1.23	454.13	5.59	$<$ .001
Random effects					
Group	Variance		SD		
Speaker (Intercept)	331.1		18.20		
Residual	520.4		22.81		

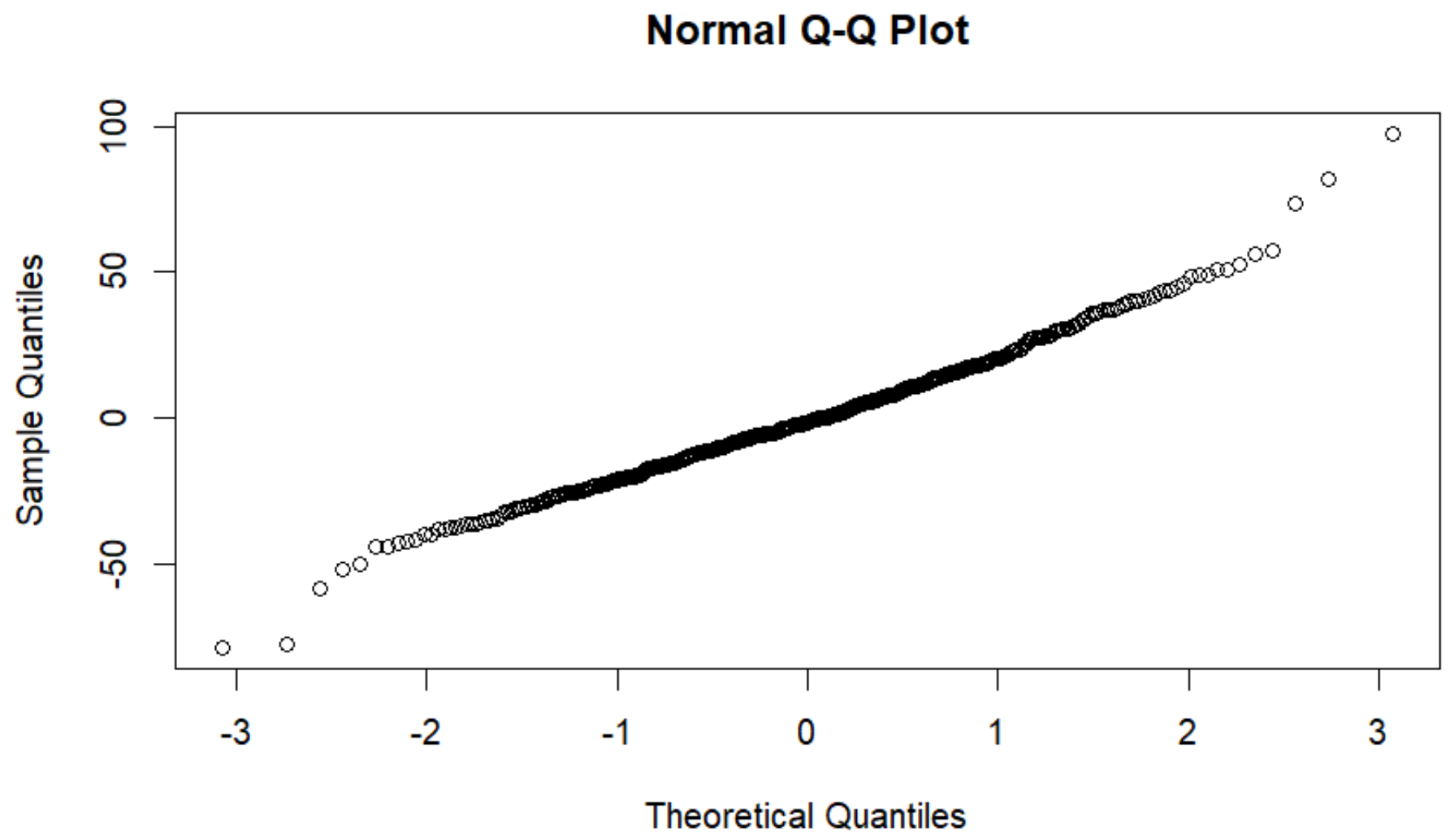

Figure S2. Q-Q plot of residuals for the vowel duration model.

1.3 Linear mixed-effects model for /st/ duration (st_dur)

Fixed effect	Estimate	SE	df	t value	p value
(Intercept)	163.25	6.87	19.58	23.77	$< .001$
Status (MC)	10.34	6.27	453.28	1.65	.100
Prosody (final)	67.67	2.47	453.02	27.37	$< .001$
log.WordFreq	-8.21	1.47	455.49	-5.57	$< .001$
VowelLength (long)	24.20	6.92	454.53	3.50	$< .001$
ClusterLength (four)	30.71	8.67	454.87	3.54	$< .001$
Status $\times$ log.WordFreq	8.87	2.02	455.18	4.39	$< .001$
Status $\times$ VowelLength	-39.64	8.42	455.96	-4.71	$< .001$
Status $\times$ ClusterLength	-42.52	9.92	455.20	-4.29	$< .001$
Random effects					
Group	Variance		SD		
Speaker (Intercept)	575.3		23.98		
Residual	727.3		26.97		

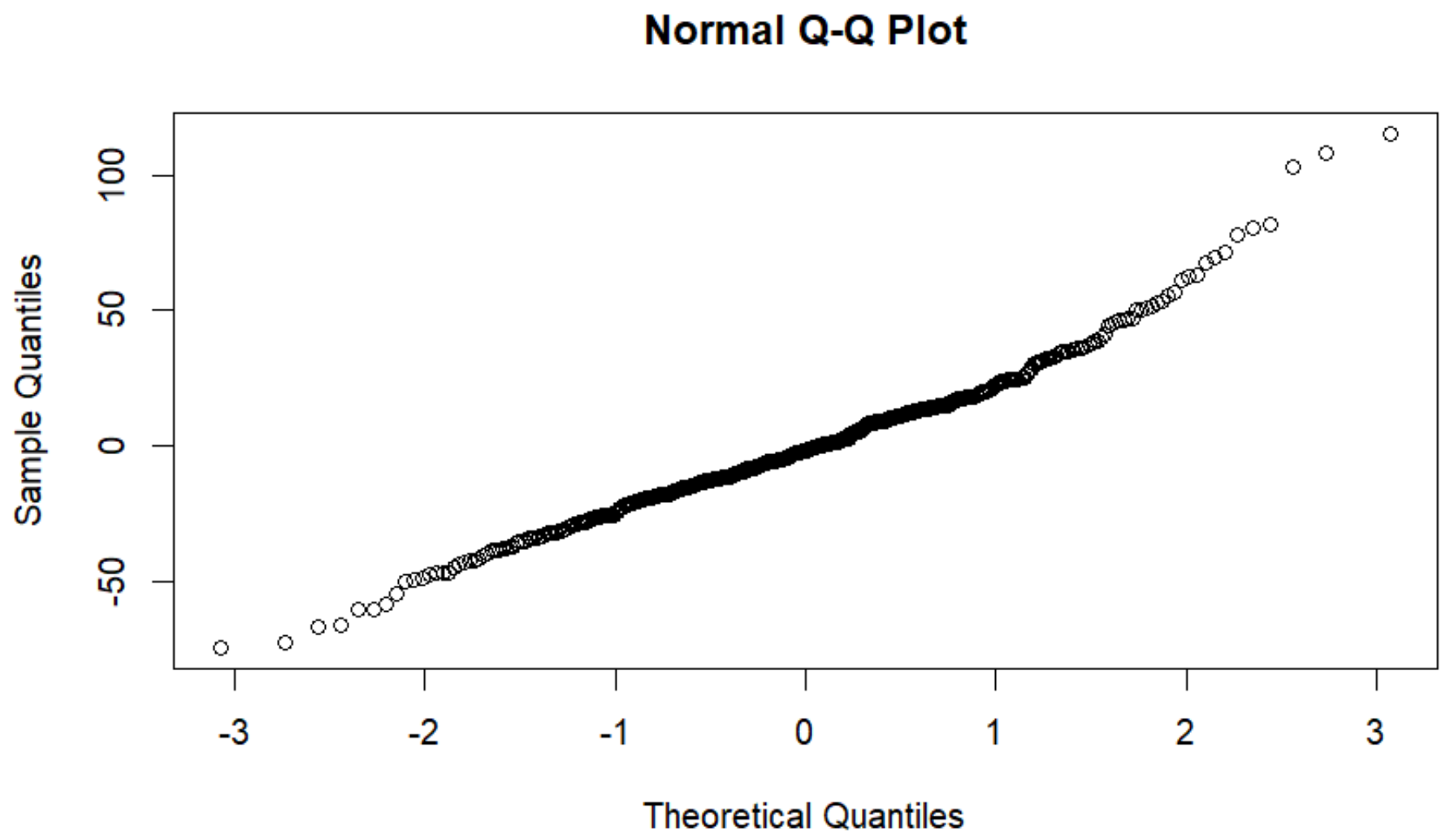

Figure S3. Q-Q plot of residuals for the /st/ duration model.

1.4 Linear mixed-effects model for /s/ intensity (s_int)

Fixed effect	Estimate	SE	df	t value	p value
(Intercept)	0.736	0.012	17.16	59.09	$<$ .001
Status (MC)	-0.021	0.010	412.26	-2.01	.045
log.WordFreq	0.010	0.002	413.65	4.28	$<$ .001
VowelLength (long)	-0.035	0.011	412.96	-3.04	.002
ClusterLength (four)	-0.061	0.014	413.21	-4.27	$<$ .001
Status $\times$ log.WordFreq	-0.015	0.003	413.17	-4.70	$<$ .001
Status $\times$ VowelLength	0.043	0.014	414.02	3.11	.002
Status $\times$ ClusterLength	0.067	0.016	413.50	4.13	$<$ .001
Random effects					
Group	Variance		SD		
Speaker (Intercept)	0.002		0.045		
Residual	0.001		0.041		

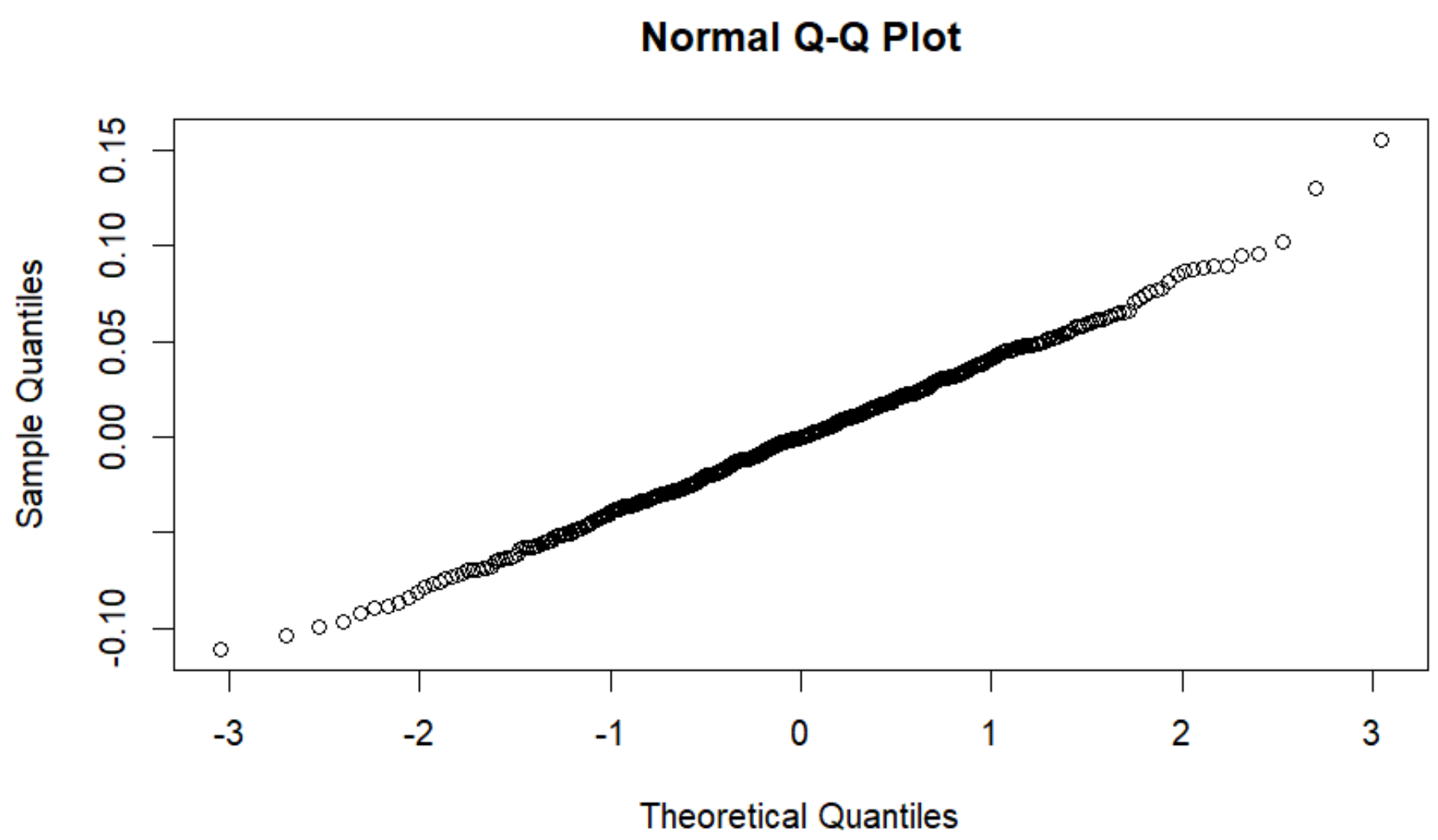

Figure S4. Q-Q plot of residuals for the /s/ intensity model.

1.5 Linear mixed-effects model for /s/ centre of gravity (s_cog)

Fixed effect	Estimate	SE	df	t value	p value
(Intercept)	7095.02	180.35	18.53	39.34	$<$ .001
Status (MC)	2352.43	307.83	411.09	7.64	$<$ .001
Prosody (final)	-316.53	58.85	411.93	-5.38	$<$ .001
log.WordFreq	125.61	42.10	415.73	2.98	.003
VowelLength (long)	-40.10	177.49	413.75	-0.23	.821
ClusterLength (four)	-408.32	225.92	414.25	-1.81	.071
Labiality (yes)	-1194.50	110.19	415.24	-10.84	$<$ .001
Status $\times$ log.WordFreq	489.18	91.76	412.06	5.33	$<$ .001
Status $\times$ VowelLength	-1896.09	361.74	412.24	-5.24	$<$ .001
Status $\times$ ClusterLength	-913.44	308.02	413.25	-2.97	.003
Status $\times$ Labiality	2097.61	299.82	411.85	7.00	$<$ .001
Random effects					
Group	Variance		SD		
Speaker (Intercept)	410427		640.6		
Residual	363011		602.5		

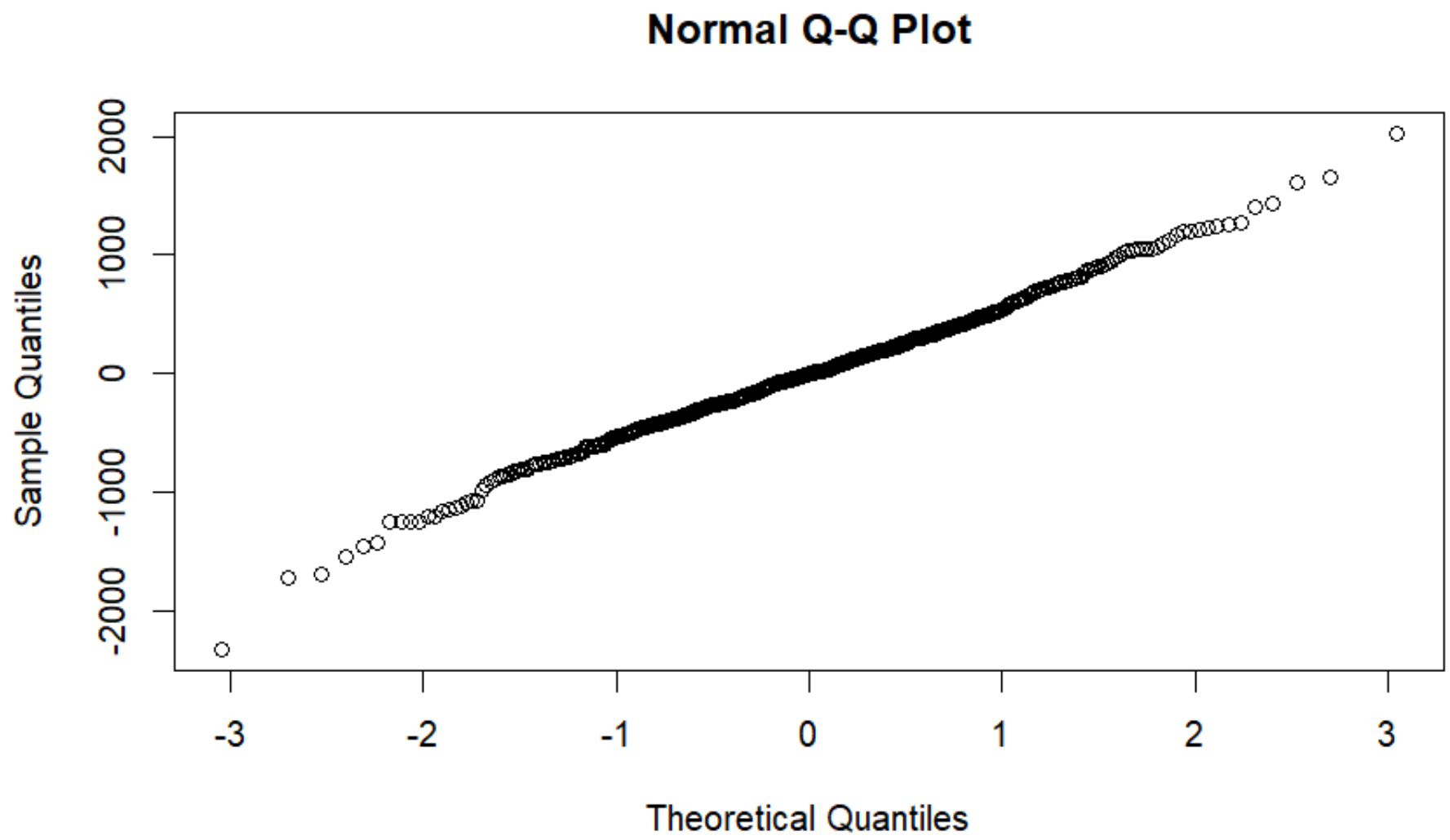

Figure S5. Q-Q plot of residuals for the /s/ COG model.
